# Salivary total tau: a clinically practical measure of tau neuropathology in Alzheimer’s disease

**DOI:** 10.1007/s00415-026-13651-1

**Published:** 2026-02-18

**Authors:** Alison R. Bamford, Christopher Logan, Quynh Theresa Do, Kelly Nguyen, Liv C. McMillan, Michael A. Yassa, William R. Shankle, Elizabeth A. Thomas

**Affiliations:** 1 Department of Neurobiology and Behavior, University of California Irvine, Irvine, CA USA; 2 Institute for Interdisciplinary Salivary Bioscience Research, University of California Irvine, Irvine, CA USA; 3 Shankle Memory Clinic, Newport Beach, CA USA; 4 Center for the Neurobiology of Learning and Memory, University of California, Irvine, Irvine, CA USA; 5 Department of Neurosciences, The Scripps Research Institute, La Jolla, CA USA

**Keywords:** Biomarker, Tau, Dementia, Neurodegenerative, Saliva

## Abstract

Neurofibrillary tangles, consisting of intracellular accumulations of the protein tau, are a hallmark feature of Alzheimer’s disease (AD), and are thought to contribute to neuronal dysfunction and death during the disease process. The quantification of tau proteins in cerebrospinal fluid (CSF) or plasma has enormous utility for AD diagnosis; however, validated non-invasive measures of tau protein are lacking and would have added value for widespread screening. In this study, we quantified the levels of total tau (t-tau), along with neurofilament light (NfL) and glial fibrillary acidic protein (GFAP), in saliva samples from 111 participants, including those with CSF biomarker-confirmed AD, mild AD, non-AD cognitively impaired (CI) and cognitively unimpaired (CU) older adults, using immunoassays on the Meso Scale Discovery platform. We find that salivary levels of t-tau were significantly elevated in AD and mild AD, but not other CI patients, compared to CU adults, while salivary levels of NfL and GFAP showed no significant differences across cohorts. In addition, we found that salivary t-tau was significantly correlated with CSF biomarker measures, including significant positive correlations with CSF t-tau and p-tau 181 (0.257; *p* = 0.016 and 0.276; *p* = 0.009 for t-tau and p-tau 181, respectively). Salivary t-tau was also found to predict AD cases compared to CU individuals with an area under the curve of 0.834 (95% CI 0.74–0.93; *p* < 0.0001). Finally, we observed that salivary t-tau levels were significantly negatively correlated with cognitive performance in AD patients, as well as all individuals together. These findings suggest that salivary t-tau might represent a non-invasive biomarker specific to AD pathology and could aid in early detection of AD or for clinical screening purposes.

**Supplementary Information:**

The online version contains supplementary material available at 10.1007/s00415-026-13651-1.

## Introduction

Alzheimer’s disease (AD), the most common cause of dementia in older adults, is characterized by the accumulation of two specific protein pathologies in the human brain: amyloid plaques, composed of extracellular amyloid-β (Aβ) peptides, and intracellular neurofibrillary tangles, composed of abnormal tau protein [1]. Accumulation of Aβ is known to precede tau accumulation in the brain, starting as early as 2 decades before the onset of memory impairment, whereby tau tangles form later in the disease process and are more closely associated with neuronal loss and cognitive impairment [2]. Tau is a microtubule-associated protein, which plays important roles in axonal transport, signal transduction, and maintaining normal morphology of neurons in the brain [3]. Accordingly, abnormal levels or forms of tau are associated with many neurodegenerative diseases and tauopathies; however, most of the tau that constitutes neurofibrillary tangles in the AD brain is in a hyperphosphorylated form [4]. Tau can be phosphorylated at many different residues, with phosphorylation at threonine 217 (p-tau 217) and threonine 181 (p-tau 181) showing the greatest diagnostic value for AD, along with measurements of total tau (t-tau) and Aβ42, in the CSF [5]. CSF sampling, however, is an invasive procedure that requires highly-trained personnel for its collection. As such, there is a need to identify non-invasive approaches for biomarker utility in clinical research studies.

Although blood measures of t-tau/p-tau have shown promise for AD diagnostics [6–8], saliva is the quintessential non-invasive biofluid that can be collected in any setting and does not require any trained personnel. Several studies using different methodologies and sample collection procedures have reported successful quantification of t-tau and different p-tau species in saliva samples. However, the results are inconsistent as to whether levels are changed in relationship to AD. Studies using either ELISA or mass spectrometry found that salivary p-tau levels were higher in AD patients compared to a control group [9, 10], as well as salivary p-tau at serine 396 [11], although other studies did not see such differences [12, 13]. Three studies focusing on different tau species in saliva found no difference between AD and control groups [12–14], except for a non-significant increase in t-tau in AD patients [15]. However, other studies have reported an elevation of the p-tau/t-tau ratio in AD patients compared to non-AD controls [11, 14]. Importantly, the diagnosis of AD patients in all of these past studies was based on neuropsychological evaluation and only one study had available CSF tau levels for their patient populations, in which only p-tau/t-tau ratios were reported [11]. In addition, different sample collection protocols were used as well as differences in pre-analytical factors, including the use of protein stabilizers and different sample processing methods. Hence, the current literature is uncertain as to role of salivary tau in AD.

To further explore the use of saliva for AD biomarker research, we quantified thd levels of t-tau, as well as two other common neurodegenerative disease biomarkers, neurofilament light (NfL) and glial fibrillary acidic protein (GFAP), in saliva samples from patients with CSF biomarker-confirmed AD, patients with cognitive impairment, and in cognitively unimpaired older adults. Further, we tested whether salivary levels of t-tau, NfL or GFAP markers were associated with Aβ42, t-tau, and p-tau, measured in CSF samples and whether salivary t-tau had any predictive value in determining AD cases.

## Materials and methods

### Participants

Cognitively unimpaired (CU) individuals, cognitively impaired (CI) individuals, and patients with AD were recruited from the Shankle Memory Clinic (Newport Beach, CA). Inclusion criteria consisted of 60–90 years of age and fluency in spoken English. Most patients underwent a lumbar puncture for diagnostic purposes. The diagnosis of AD was based on the CSF Skillback criteria [16], CSF Aβ42/p-tau 181 ratio < 6.5, and t-tau > 350 pg/ml, which have a 95% sensitivity and 87% specificity for AD diagnosis [16]. This is consistent with the recently determined first-line criteria determined for AD diagnosis [17]. Patients with mild AD pathology (“Mild AD”) were classified according to their CSF AD biomarker profile, having an Aβ42/p-tau 181 ratio < 6.5 and t-tau < 350 pg/ml. In addition, a clinical diagnosis of AD was given to two patients based on amyloid PET findings and for an additional patient based on neuropsychological assessment. Cognitively related functional severity was assessed using the Functional Assessment Staging Test (FAST) [18]. CI patients had a FAST stage of 3 or higher and did not meet CSF biomarker criteria for AD, per above. An additional two individuals, who did not provide a CSF sample and were not diagnosed with AD based on clinical assessments, were also included. The CI group consisted of patients with different clinical conditions, including cerebrovascular disease, cerebral amyloid angiopathy, tauopathy, multisystem atrophy, cerebral ischemia, frontal temporal lobe dementia, or lacunar infarcts. CU patients had a FAST stage of no greater than 2 and did not meet CSF biomarker criteria for AD. All experimental protocols were approved by the Institutional Review Board (IRB) of the University of California, Irvine, and all subjects provided written informed consent for each study.

### Saliva collection

All donors were asked to refrain from smoking, eating, drinking, or oral hygiene procedures for at least 1 h prior to samples collection. Saliva samples were collected between 8:30 am and 5 pm using the passive drool method according to our previous studies [19, 20]. For cognitively unimpaired subjects, the exact time of the sample collection was recorded. Roughly one to two milliliters of unstimulated whole saliva was obtained within a 5 min period. Samples were immediately frozen at − 20C at the time of collection, then stored at − 80C for long-term storage. At the time of use, saliva samples were thawed and centrifuged (5000*g*; 10 min; 4C) to remove mucins, insoluble material, and cellular debris. Supernatants were collected and used for all assays.

### Biomarker measurements in saliva

Levels of GFAP, NfL, and t-tau were quantified in saliva samples using electrochemiluminescence immunoassay kits from Meso Scale Discovery [(MSD), Gaithersburg, MD, U-PLEX, 3-plex for GFAP, NfL and t-tau] in a 96-well format. Wells were first coated with unique biotinylated capture antibodies for 1 h at RT. Saliva samples (25 μl) were diluted with 25 μl Diluent 12 containing 1X Complete Protease Inhibitor (Sigma-Aldrich) and 1 mM EDTA. Assays were carried out according to the manufacturer’s protocol, except with an extended incubation time of 2 h. Whenever possible, samples were assayed after a single thaw. On each platform, a single batch of reagents was used for all samples. Internal control samples consisting of three plasma samples had been aliquotted into ten tubes. Measurements were performed in duplicate, and sample measurements were accepted if coefficients of variation across duplicates were < 20%. Concentrations (pg/ml) for each protein were determined with MSD Discovery Workbench Software using curve fit models. Lower limits of detection (LLoD) were calculated as the concentration corresponding to the signal 2.5 times standard deviation above background. Intra-assay CVs were determined by taking the mean signal CV across each plate and were between 4.7 and 9.9% for all markers. The inter-assay CV was determined using the mean concentration of three internal control plasma samples that were run on each plate and were 11.9%, 22.4%, and 13.2% for NfL, GFAP, and t-tau, respectively. Detection rates were 80.5%, 97.7%, and 100%, for NfL, GFAP and t-tau respectively, respectively. Salivary markers that were below detection were not included in any analysis. Total protein levels in each saliva sample were determined using the BCA assays (Pierce™) according to previous studies [21].

### CSF AD biomarker measures

Levels of Aβ42, t-tau, and p-tau 181 were quantified in CSF samples from patients who were undergoing a lumbar puncture for diagnostic purposes. These biomarkers were quantified by ELISA (Athena Neurosciences).

### Cognitive assessments

The 3-min, F-A-S test of phonemic verbal fluency (common noun naming, 1 min per letter) was administered to all recruited subjects. Scores analyzed were the total number of words named in 3 min and the number of common nouns correctly named, not including repetition errors.

### Statistical analyses

All statistical analyses were performed using RStudio R 4.3.1, IBM® SPSS® Statistics (version 25) or contchart.com. Raw data were first tested for normality using the Shapiro–Wilk normality test and analyzed accordingly using parametric or non-parametric tests. An outlier analysis was performed on the raw data using Iglewicz and Hoaglin’s robust test for multiple outliers (two-sided test, modified *Z* score ≥ 5) using Ln-transformed data. Two low outliers for t-tau were identified in the AD cohort and these were excluded in the statistical analyses. Two high outliers were identified in the GFAP dataset, and these were omitted from statistical analysis. Comparisons of saliva biomarkers across groups were determined using Kruskal–Wallis, with post hoc tests determining significant differences of each cohort compared to the CU group. Partial correlation analysis adjusting for age and sex was used to compare saliva biomarker data to CSF biomarker levels and was carried out using a non-parametric adjustment. A Bonferroni correction was applied to the results from our partial correlations analysis to adjust for multiple comparisons. The ability of salivary t-tau to identify CSF biomarker-confirmed AD cases was determined using logistic regression models and receiver operating characteristic (ROC) curve analysis.

## Results

### Saliva measures of NfL, GFAP, and t-tau

In the current study, we recruited *n* = 111 subjects, who were grouped into four cohorts: AD, mild AD pathology, CI, and CU, using the criteria described in the Methods (see Table 1). There were no significant differences in sex distribution or year of education across the groups; however, the CU cohort was significantly younger than the other three dementia cohorts (Kruskal–Wallis test, *p* < 0.0001) (Table 1). The number of patients carrying at least one *APOE4* allele was significantly higher in the AD patients compared to the other groups (Chi-square test, *p* = 0.0003) (Table 1).

**Table 1:** Summary of participants used in this study

	AD	Mild AD	Other CI	CU	*p* value
Number	59	11	20	21	
Male:female	30:29	5:6	10:10	12:7	n.s.
Mean age in years (S.D.)	77.6 (5.7)	75.2 (8.5)	79.1 (6.7)	70.35 (10.2)	***
Edu (years)	16.6	16.2	15.9	17.3	n.s.
% *APOE4*(+)	80.0%(+)	45.4%(+)	29.4%(+)	42.1%(+)	***

*AD* Alzheimer’s disease, *CI* cognitively impaired, *CU* cognitively impaired, *APOE* apolipoprotein E, show % of those carrying at least one *APOE4* allele

*p* values reflect significant differences across the different groups. Differences in age were determined using Kruskal–Wallis test and differences in %*APOE4* positivity were determined using Chi-square test. *n.s.* not significant; ***, *p* < 0.001

Using MSD immunoassays, we quantified the levels of NfL, t-tau, and GFAP in saliva samples. A summary of the mean and median biomarker levels within each group is shown in Table 2. No salivary biomarker showed a significant correlation with age nor sex in any cohort (Table 2). Total protein present in each sample was also measured and only t-tau within the CI cohort showed a significant association with total protein in the sample (*r* = 0.514; *p* = 0.012) (Table 2).

**Table 2:** Summary of t-tau, NfL, and GFAP levels in measured saliva samples and the effects of each biomarker with age, sex, and salivary total protein levels

	Total tau			
	AD	Mild AD	Other CI	CU
*N*	59	11	20	21
Mean ± S.D. (pg/ml)	42.95 ± 43.40	34.145 ± 33.46	22.54 ± 19.39	11.35 ± 7.3
Median (range) (pg/ml)	25.87 (0.89–204.4)	23.88 (6.23–128.62)	15.99 (0.58–74.41)	8.97 (1.34–25.6)
Age (*r*; *p* value)	0.012; 0.930	0.123; 0.718	0.219; 0.301	(−)325; 0.162
Sex (*p* value)	0.457	0.097	0.973	0.759
Total protein (*r*; *p* value)	0.183; 0.141	0.407; 0.150	0.514; 0.012	0.082; 0.489

	NfL			
	AD	Mild AD	Other CI	CU
*N*	46	10	15	19
Mean ± S.D. (pg/ml)	10.02 ± 7.85	5.64 ± 5.37	15.71 ± 18.72	12.61 ± 12.0
Median (range) (pg/ml)	7.38 (0.378–23.85)	3.73 (0.579–16.16)	12.06 (0.233–86.29)	7.51 (1.52–51.21)
Age (*r*; *p* value)	0.038; 0.809	(−)0.201; 0.576	(−)0.274; 0.255	0.424; 0.079
Sex (*p* value)	0.063	0.755	0.142	0.467
Total protein (*r*; *p* value)	(−)0.043; 0.759	0.378; 0.227	0.276; 0.266	0.195; 0.180

	GFAP			
	AD	Mild AD	Other CI	CU
*N*	58	10	19	20
Mean ± S.D. (pg/ml)	31.75 ± 39.01	25.55 ± 22.67	24.54 ± 27.4	48.54 ± 37.32
Median (range) (pg/ml)	17.15 (0.817–159.7)	15.96 (1.961–59.35)	16.17 (0.689–114.1)	42.63 (1.72–122.6)
Age (*r*; *p* value)	(−)0.014; 0.920	0.218; 0.542	(−)0.334; 0.119	(−)0.226; 0.338
Sex (*p* value)	0.577	0.534	0.529	0.505
Total protein (*r*; *p* value)	(−)0.043; 0.731	0.557; 0.094	0.094; 0.681	0.070; 0.762

Age and total protein associations were determined using Spearman correlation analysis. Sex associations were determined by Mann–Whitney *U* test

*AD* Alzheimer’s disease, *CI* cognitively impaired, *CU* cognitively impaired, *N* number of samples measured within the detection limit, *S.D.* standard deviation

### Comparisons of salivary biomarkers across cohorts

**Fig. 1:**
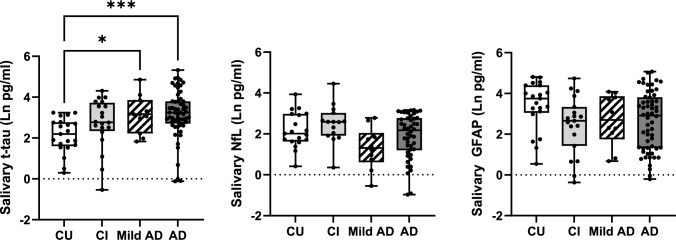
Comparisons of salivary t-tau, NfL, and GFAP across different groups. Alzheimer’s disease (AD); cognitively impaired (CI); cognitively impaired (CU). Asterisks denote significant differences between the indicated groups according to Kruskal–Wallis test, multiple comparison post hoc test comparing each group to the CU group

Comparing salivary biomarkers across cohorts, we found a significant difference in salivary t-tau levels (Kruskal–Wallis, *H*(4) = 19.38; *p* = 0.0002), with Dunn’s multiple comparison post hoc test showing a significant difference between patients with AD from CU (*p* < 0.0001) and those with Mild AD versus CU subjects (*p* = 0.031) (see Fig. 1). In contrast, we did not observe significant differences in levels of salivary GFAP nor NfL across cohorts (Fig. 1), except for a non-significant decrease in salivary GFAP and NfL in mild AD patients compared to CU individuals (Table 2, Fig. 1). Given the correlation between salivary t-tau and total protein in the CI group, we also compared salivary t-tau normalized to total protein levels from each sample and observed a similar significant difference across groups (Kruskal–Wallis test, *H*(4) = 13.98; *p* = 0.003), with post hoc comparisons showing a significant increase in the AD patients compared to the CU individuals (*p* < 0.001) (Suppl Fig. 1).

### Associations between saliva and CSF tau measures

**Fig. 2:**
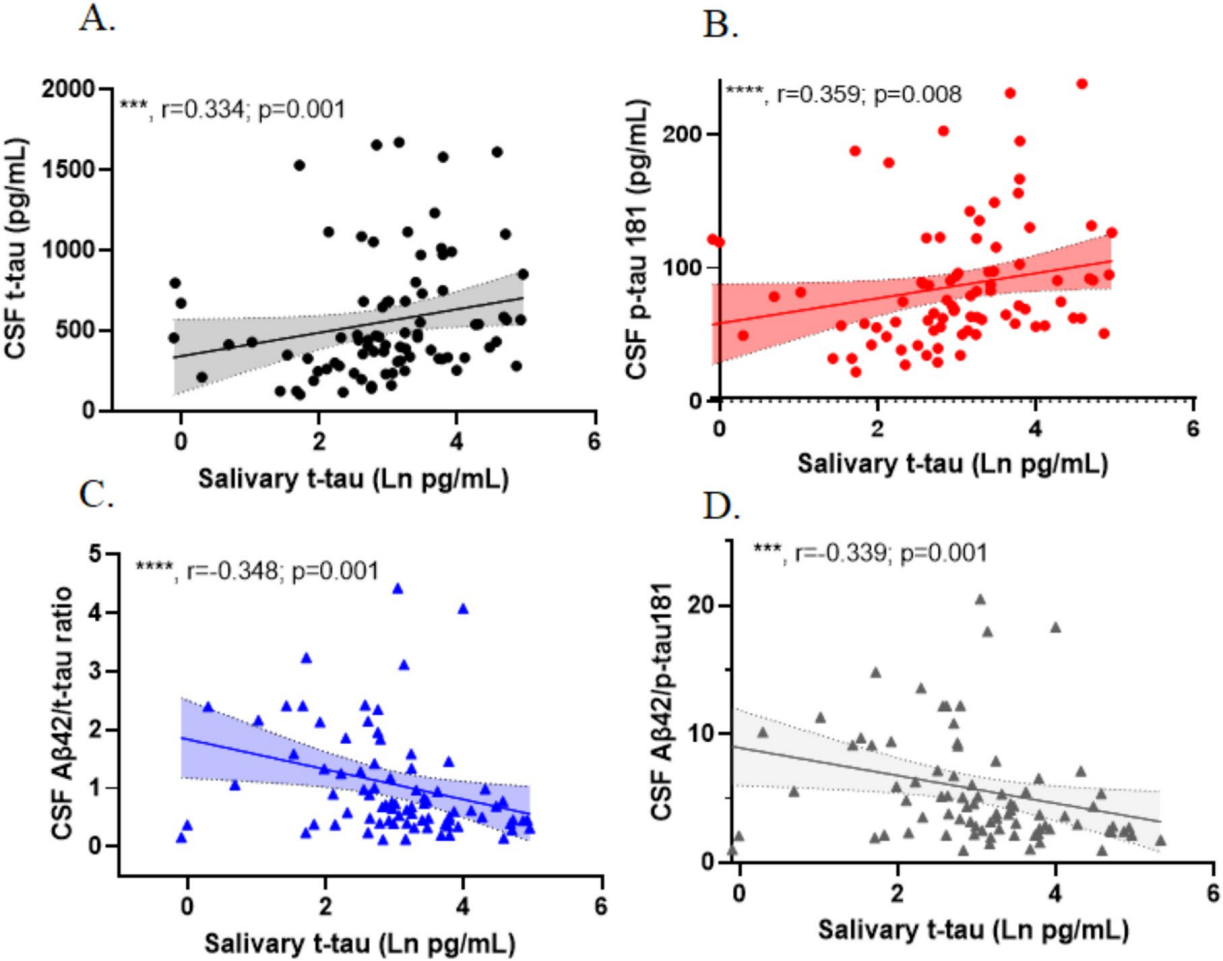
Correlations between salivary t-tau and AD biomarkers measured in CSF samples from the same individuals. Unadjusted Spearman correlations are shown with the indicated correlation coefficients (rho, *r*) and *p* values. Data points only reflect values that were above the detection limit of the assay for each biomarker

Next, considering only those patients who had available CSF measurements of Aβ42, t-tau, and p-tau 181 (*n* = 91; Suppl Table 1), we tested for correlations between salivary t-tau and CSF levels of Aβ42, t-tau, and p-tau 181, as well as the ratios among these biomarkers using partial correlation analyses, adjusting for age and sex. Before and after adjusting for age and sex, we found statistically significant positive correlations between salivary t-tau and both CSF t-tau and p-tau 181 (0.297; *p* = 0.004 and 0.321; *p* = 0.009 for t-tau and p-tau 181, respectively, age- and sex-adjusted) (Table 3, Fig. 2). Significant negative correlations between salivary t-tau and the ratios of Aβ42/t-tau and Aβ42/p-tau 181 were observed, also before and after adjusting for age and sex (see Table 3, Fig. 2). No significant correlations were observed between salivary NfL and GFAP and any CSF biomarker measure (Table 3).

**Table 3:** Partial correlations between AD biomarkers measured in salivary and CSF biofluids

Unadjusted correlations			CSF					
			Aβ42	p-tau 181	t-tau	Aβ42/p-tau 181 ratio	Aβ42/t-tau ratio	p-tau 181/t tau ratio
Saliva	t-tau (pg/ml)	Rho	0.174	**0**.**359**	**0**.**334**	− **0**.**339**	− **0**.**348**	− **0**.**229**
		*p*-value	0.1	**0**.**008**	**0**.**001**	**0**.**001**	**0**.**001**	**0**.**028**
	NfL (pg/ml)	Rho	0.2	0.032	0.034	0.134	0.116	0.064
		*p*-value	0.09	0.787	0.778	0.26	0.328	0.59
	GFAP (pg/ml)	Rho	0.147	− 0.036	0.004	0.108	0.058	− 0.066
		*p*-value	0.173	0.738	0.97	0.318	0.593	0.546

Age- and sex-adjusted			CSF					
			Aβ42	p-tau 181	t-tau	Aβ42/p-tau 181 ratio	Aβ42/t-tau ratio	p-tau 181/t tau ratio
Saliva	t-tau (pg/ml)	Rho	− 0.148	**0**.**321**	**0**.**297**	− **0**.**311**	− **0**.**325**	− 0.194
		*p* value	0.169	**0**.**009**	**0**.**004**	**0**.**006**	**0**.**004**	0.07
	NfL (pg/ml)	Rho	0.181	0.064	0.062	0.099	0.08	0.048
		*p *value	0.131	0.598	0.606	0.413	0.506	0.69
	GFAP (pg/ml)	Rho	0.118	− 0.045	0.003	0.102	0.052	− 0.081
		*p *value	0.281	0.68	0.979	0.352	0.634	0.459

Unadjusted correlations reflect Spearman correlations. Age- and sex-adjusted correlations were carried out using partial correlations with a non-parametric adjustment. Bold font indicates those correlations that were statistically.

### Salivary t-tau levels can predict AD diagnosis

**Fig. 3:**
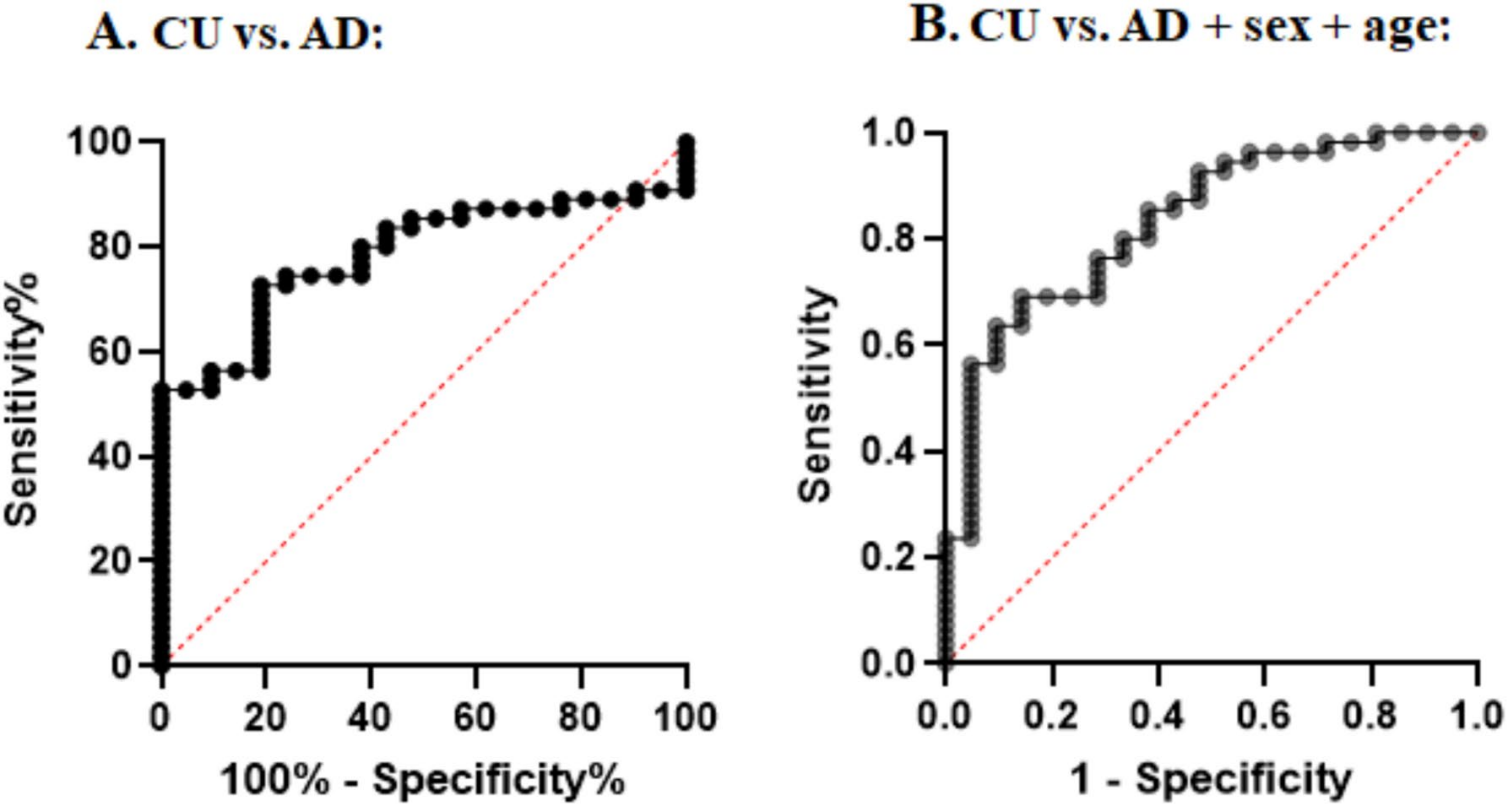
Salivary t-tau can predict AD cases compared to cognitively unimpaired (CU) individuals. Panel **A** shows the results from ROC analysis, AUC = 0.782; 95% CI 0.682–0.885; *p* = 0.0001. Panel **B** shows the results of logistic regression analysis, including age and sex as co-variables with AUC = 0.834; 95% CI 0.736–0.933; *p* < 0.0001

Next, we performed receiver operating characteristic (ROC) analysis to determine if salivary t-tau could predict AD cases compared to CU individuals. We found that salivary t-tau can predict CSF-confirmed AD cases with an area under the curve (AUC) of 0.785 (95% CI 0.682–0.885; *p* = 0.0001) and a cut-off of 16.5 pg/ml with 72.7% and 80.95% sensitivity and specificity, respectively (Fig. 3). Using logistic regression analysis to include age and sex in the model, the AUC improved to 0.834 (95% CI 0.736–0.933; *p* < 0.0001) (Fig. 3). Adding *APOE4* gene status to the model was confounded by the fact that most of the AD cases carried at least one *APOE4* allele, and hence is not shown.

### Salivary t-tau levels are associated with cognitive performance

**Fig. 4:**
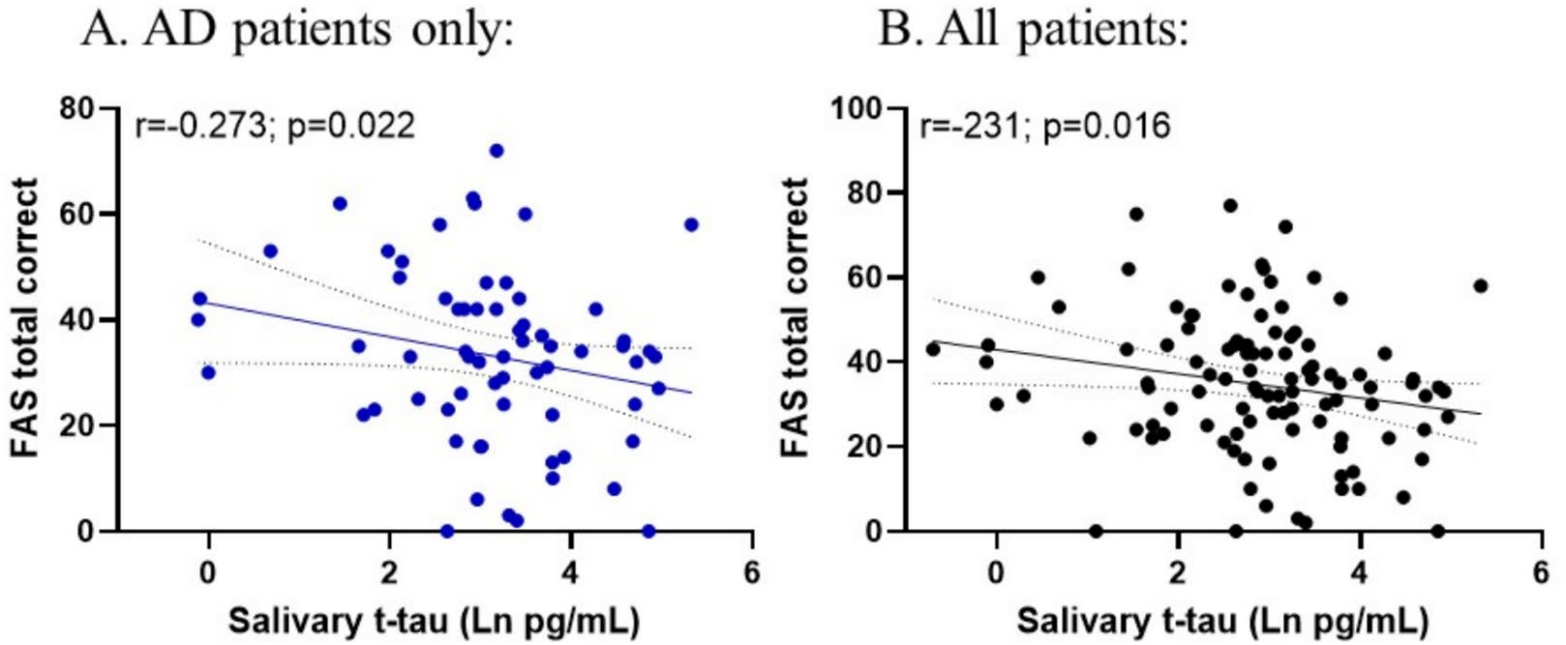
Correlation between salivary t-tau and F-A-S word test in AD patients (**A**) and all patients (**B**). Correlations reflect Spearman correlations from AD patients (including those who with mild AD pathology) (*n* = 66) and all patients (*n* = 107)

Salivary t-tau levels were compared to F-A-S cognitive test performance. After adjusting for age and sex, salivary t-tau correlated negatively with the number of unique, common nouns named in all AD patients (*r* = − 0.273; *p* = 0.022; *n* = 66) and in all patients who could perform cognitive testing (*r* = − 0.231; *p* = 0.016; *n* = 107) (Fig. 4).

## Discussion

In this study, we found that t-tau levels measured in saliva were significantly higher in patients with AD and those with mild AD pathology compared to CU subjects. Further, we found that salivary t-tau levels were significantly correlated with CSF AD biomarker levels and could predict AD diagnosis relative to CU subjects. Salivary t-tau levels were not elevated in other CI patients who were CSF AD biomarker-negative, suggesting that salivary t-tau is reflecting AD-related pathology in the brain. Non-invasive biomarkers for AD would have enormous value for widespread screening efforts, and our studies would suggest that salivary t-tau might serve in this manner.

One major difference in our study, compared to previous studies assessing levels of tau proteins in saliva in the context of AD [9–15], was the use of CSF biomarker-confirmed AD cases with documented AD pathology (Skillback criteria [16]). This allowed us to directly assess the relationship between levels of t-tau measured in saliva and known tau pathology in the brain. Not only did we observe a significant increase in salivary t-tau in AD cases compared to CU individuals, but we also observed significant correlations between salivary t-tau and both CSF t-tau and p-tau 181 levels. Only one prior study in the saliva tau literature had access to matching saliva-CSF data, where levels of p-tau at threonine 181 and serine 396 and 404 were quantified [11]. In that study, only the p-tau/t-tau ratios were reported, hence direct comparisons to our study could not be done. Nonetheless, no significant correlations between the CSF p-tau/t-tau ratios and the salivary p-tau/t-tau ratios were observed, although a significant increase in the p-tau 396/t-tau ratio was observed in AD patients compared to normal elderly controls [11]. The diagnosis of AD in the other studies was based on neuropsychological test performance only.

Therefore, the discrepancy between the findings of our study and those of previous studies [12–14] evaluating salivary t-tau differences between AD and non-AD patients may have been due to inaccurate classification of AD or non-AD pathology when diagnoses are based on neuropsychological testing.

We did, however, observe that salivary t-tau levels were significantly negatively correlated with the number of correctly named, common nouns in both AD patients as well as in the entire sample (AD and Non-AD). Because many neurodegenerative disorders have elevated total tau pathology, a salivary t-tau test may be a useful measure of its presence and amount, regardless of the neurodegenerative etiology.

While t-tau and p-tau in the CSF originate from the brain, the origin of tau species in saliva is uncertain. It is possible that tau species present in saliva could come directly from the brain. Two scenarios could reflect this concept. First, tau proteins secreted by neuronal cells into the extracellular space would have direct communication with the CSF which can exit the brain via various routes. For example, the cribriform plate, a bony structure separating the cranial and nasal cavities, provides a passage for CSF outflow along the olfactory nerve bundles [22]. Periaxonal transport via this route could allow tau to be present in the oral cavity ending up in whole saliva. Second, it is possible that tau proteins could be released from the nerve endings of nerve fibers that innervate salivary glands. Two major cranial nerves fit this role, with the facial nerve (cranial nerve VII) directly innervating the submandibular and sublingual glands, and the glossopharyngeal nerve (cranial nerve IX) directly innervating the parotid gland.

A potential peripheral source of tau proteins could be the salivary glands themselves. For example, both tau and p-tau proteins have been detected in the submandibular gland using Western blot analysis [23]. In this situation, salivary tau levels could directly or indirectly be reflecting pathological changes occurring in the AD brain.

One thing to note is that levels of t-tau measured in saliva on our MSD platform in this study were robust and detectable in 100% of subjects (10–45 pg/ml across groups), compared to CSF (100–1600 pg/ml). We do not believe that salivary t-tau is derived from blood, given that levels are much lower in blood [20], and plasma levels of t-tau do not correlate with t-tau levels in the CSF [20, 24]. Also, the large size of tau would likely prevent passive diffusion from blood into saliva. Further investigations are warranted to fully understand the potentially complex tau dynamics in the brain and periphery.

Regarding the other biomarkers of neurodegeneration measured in this study, we did not observe significant differences in the levels of salivary GFAP, nor NfL, across cohorts. However, non-significant decreases in salivary GFAP and NfL were observed in mild AD patients compared to CU individuals. These findings are consistent with our previous studies that showed a significant decrease in salivary NfL levels in patients with Huntington’s disease compared to normal controls, a finding that is opposite to what it observed when NfL levels are measured in plasma [20].

In addition to anti-amyloid treatments [25–27], anti-tau therapies represent one of the most active fields of current AD therapeutic research [28]. If sensitive and quantitative, a non-invasive biomarker of tau neuropathology would have applications in detecting tau neuropathology early in the disease process, for measuring the degree to which putative treatments could lower tau in the brain, and to determine how such lowering of tau alters the cognitive function. While more expensive measures of tau pathology, such as Tau-PET imaging and CSF tau, are useful for scientific research [29], they are pragmatically and economically unsuited for assessing tau neuropathology in patient populations. Future studies could compare salivary tau biomarkers to the new tau blood biomarkers (in particular, p-tau 217), as they could prove complementary or ultimately provide different snapshots of the neurodegenerative pathology.

The findings of this study also indicate that salivary t-tau correlates with at least one measure of cognitive function—phonemic fluency—regardless of neurodegenerative pathology. This correlation implies a direct relationship between salivary and central nervous system tau pathology. This is very useful because one may be able to use salivary t-tau and cognitive testing to assess the degree of tau pathology in the brain. As treatments become available, a brief cognitive test and salivary t-tau could measure the relation between reduced tau pathology and change in cognitive function and the relation between. If salivary t-tau is sensitive to tau neuropathology, it may also be used to detect early or preclinical AD, allowing treatments to be given at a time most apt for disease modification.

## Supplementary Information

Below is the link to the electronic supplementary material.

Supplementary file1 (DOCX 35 KB)

## Data Availability

Anonymized summary data will be shared by reasonable formal request from qualified researchers, subject to a data sharing agreement and in compliance with the requirements of the funding bodies and institutions.

## Declarations

### Generative AI and AI-assisted technologies in the writing process

No AI of any kind was used in preparing this manuscript.
