# Supplementary material for: Salivary total tau: a clinically practical measure of tau neuropathology in Alzheimer’s disease
Source: J Neurol. 2026 Feb 18;273(2):149. doi: 10.1007/s00415-026-13651-1 (PMC12917055; doi:10.1007/s00415-026-13651-1)
Supplement: Supplementary file 1 — Supplementary file1 (DOCX 35 KB) [file 415_2026_13651_MOESM1_ESM.docx]

**Suppl Figure 1. Comparison of salivary t-tau across different cohorts when normalized by total protein.**

Alzheimer’s disease (AD); cognitively impaired (CI); cognitively impaired (CU). Asterisks denote significant differences between the indicated groups according Kruskal-Wallis test multiple comparison post-hoc test comparing each group to the CU group.
